# The E3 Ubiquitin Ligase SYVN1 Plays an Antiapoptotic Role in Polycystic Ovary Syndrome by Regulating Mitochondrial Fission

**DOI:** 10.1155/2022/3639302

**Published:** 2022-09-19

**Authors:** Lihua Sun, Hongjuan Ye, Hui Tian, Lirong Xu, Junjie Cai, Caixia Zhang, Rongxiang Wang, Hui Yang, Shuang Zhao, Jiaozhi Zhang, Shaorong Gao

**Affiliations:** ^1^Department of Reproductive Medicine Center, Shanghai East Hospital, School of Life Sciences and Technology, Tongji University, Shanghai, China; ^2^Department of Reproductive Medicine Center, Shanghai East Hospital, Tongji University School of Medicine, Shanghai, China; ^3^Institute for Regenerative Medicine, Shanghai East Hospital, Shanghai Key Laboratory of Signaling and Disease Research, Frontier Science Center for Stem Cell Research, School of Life Sciences and Technology, Tongji University, Shanghai, China

## Abstract

Polycystic ovary syndrome (PCOS) is one of the most common hormonal disorders among premenopausal women. PCOS is accompanied by many other reproductive, endocrinal, and metabolic disorders thus amassing the difficulties encountered by the women affected. However, there is limited information on its molecular etiology. Synoviolin (SYVN1) is an E3 ubiquitin ligase that is thought to participate in the pathology of PCOS. However, the expression and function of SYVN1 in PCOS are unknown. In this study, we found that downregulation of SYVN1 expression was followed by increased apoptosis in the granulosa cells (GCs) of patients with PCOS. Subsequent in vitro experiments indicated that the overexpression of SYVN1 inhibited apoptosis and mitochondrial fission. Furthermore, using immunoprecipitation and western blotting, we identified that SYVN1 promoted the degradation of Drp1 via the proteasome-dependent pathway. Additionally, we generated a PCOS model in female Sprague Dawley rats and treated them with an SYVN1 inhibitor, LS-102. We observed that the inhibition of SYVN1 increased Drp1 levels and exacerbated the degeneration of GCs in the PCOS rat model. Finally, *in vitro* and *in vivo* experiments showed that SYVN1 inhibits apoptosis and mitochondrial fission by promoting Drp1 degradation in GCs. These results highlight the function of SYVN1 in PCOS and provide a potential target for the clinical treatment of PCOS.

## 1. Introduction

Polycystic ovary syndrome (PCOS) is a common disorder in women with a prevalence of 8 to 13% among those of premenopausal age [1]. PCOS can have long-term consequences such as an increased risk for cardiovascular, metabolic, and other physiological conditions that may result in a lifetime of complications [2]. PCOS also contributes to around 75% of anovulatory infertility and is involved in impaired follicular maturation, anovulation, and biochemical pregnancy [3]. Therefore, the complex mechanisms behind PCOS need further characterization and understanding.

For a cellular system to function efficiently, the proper regulation of mitochondrial dynamics is critical. Mitochondria are characterized as semiautonomous organelles that constantly undergo fusion and fission [4]. Dysregulation of fusion and fission processes could lead to many complications such as neurodegenerative, cardiovascular, and renal diseases [5, 6]. It has also been widely accepted that mitochondrial complications may contribute to other diseases, especially in situations where the molecular etiology of the disease has not been well explored. Recently, studies have implicated that a reduction in mitochondrial mass and its subsequent dysfunction contributes to the development of PCOS [7].

Granulosa cells (GCs) are the principal cells in the ovary that provide the support and environment for the developing oocyte. These cells further associate with female gametes and continue their interaction until primary and secondary oocyte maturation. Cumulus GCs transport the oocyte to the oviduct, and the mural GCs remain in the ovary to form luteal cells. These luteal cells are essential for implantation and zygote development [8]. Hence, GCs are necessary for appropriate follicular development, ovulation, steroidogenesis, and atresia [9, 10]. Interestingly, previous studies have indicated that the apoptosis of GCs contributes to abnormal folliculogenesis, anovulation, and other complications in PCOS [11]. However, it is important to note that one important source of this apoptosis in GCs is the dysregulation of mitochondrial fission. Specifically, a study on PCOS in an androgenized rat model displayed an upregulation of the key mitochondrial fission marker, dynamin-related protein 1 (Drp1) [12]. Furthermore, this upregulation was associated with increased mitochondrial fission that contributed to apoptosis and autophagy in the GCs at the antral stage of development in the PCOS model. However, this response could be partially rescued in rats with the use of exogenous gonadotropin [12].

Protein regulation and recycling is an important process carried out by the ubiquitination-proteasomal pathway. Previously, a study using protein-protein interaction network analysis showed that synoviolin (SYVN1) may be associated with PCOS [13]. SYVN1 is an E3-ubiquitin ligase protein predominantly studied in the endoplasmic reticulum- (ER-) associated degradation of cells and accumulated misfolded proteins [14]. A study by Ge et al. observed that upregulated SYVN1 could significantly inhibit apoptosis in osteoarthritis [15]. However, the role that SYVN1 plays in PCOS is not well elucidated.

In this study, we initially observed that SYVN1 is highly downregulated in the GCs of PCOS patients. Additionally, using a human ovarian granulosa-like tumor (KGN) cell line, we also confirmed that downregulated SYVN1 is associated with increased apoptosis and increased mitochondrial fission. Using both *in vitro* and *in vivo* rat models, we have found that SYVN1 modulates apoptosis in GCs through the degradation of Drp1 and the downregulation of mitochondrial fragmentation and apoptosis. This is the first study, to our knowledge, that clearly illustrates the mechanistic role of SYVN1 in PCOS. Additionally, our study provides valid therapeutic targets for the development of potential treatment strategies for PCOS.

## 2. Methods and Materials

### 2.1. Patients

All samples for this study were collected from women who were referred to the Reproductive Medicine Center, Shanghai East Hospital. Informed consent was obtained from each study participant. Follicular fluid was collected from women (*n* = 60) either undergoing in vitro fertilization (IVF) or intracytoplasmic sperm injection. Participants were divided into control (*n* = 30) and PCOS (*n* = 30) groups. PCOS patients were enrolled based on the Rotterdam criteria as mentioned in a previous study [16]. In the PCOS group, patients exhibited two of three characteristic pathologies: (a) oligomenorrhea or anovulation, (b) clinical or biological hyperandrogenism, or (c) polycystic ovary. The control group comprised women who displayed typical menstrual cycles and were undergoing IVF not due to the abovementioned reasons but due to male infertility or tubal infertility disorders. The study was performed in accordance with the Declaration of Helsinki, and ethical clearance was obtained from the ethics committee of the Shanghai East Hospital.

### 2.2. Isolation and Collection of Granulosa Cells

The follicular fluids from patients were collected and stored at 37°C until use. Initially, the cells were centrifuged at 2200 × g for 30 min at room temperature for separation from the supernatant. The cells were further suspended in 1 ml phosphate-buffered saline (PBS) and overlayed on 1 ml of Ficoll buffer (Tianjin Haoyang Biological Products Technology Co., Ltd., Tianjin, China) and centrifuged at 1000 × g for 3 min at room temperature. The GCs present at the interphase were carefully collected, washed, counted, and seeded for subsequent experiments.

### 2.3. KGN Cell Culture, Transfection, and Treatment

The human ovarian granulosa-like tumor (KGN) cell line was purchased from Procell Life Science &Technology Co., Ltd. These cells were selected because they are an ovarian granulosa-like tumor cell line with similar steroid activity and FSH receptor expression as normal GCs. The KGN cells were cultured in DMEM/F12 (Gibco, Grand Island, NY, USA) supplemented with 10% FBS (Gibco), 2 mM glutamine, streptomycin (100 mg/mL; Gibco), and penicillin (100 U/mL; Gibco). The cells were further maintained at 37°C with 5% CO_2_. KGN cells were transfected with either an empty plasmid or SYVN1 overexpression plasmids for overexpression studies or with control siRNA, SYVN1 siRNA, and Drp1 siRNA for silencing experiments (GenePharma, China). All transfection experiments were performed using Lipofectamine 2000 reagent (Invitrogen, 11668–019). After 24 h of transfection, the cells were collected for subsequent experiments. To assess the role of SYVN1, KGN cells were treated with 20 *μ*M of MG132 (Sigma-Aldrich, St. Louis, MO, USA) for 8 h and the cellular lysates were assessed for protein expression using western blotting. To assess the half-life of Drp1, KGN cells were treated with 100 *μ*g/ml cycloheximide (CHX, Calbiochem, San Diego, CA, USA) for the indicated hours. Subsequently, total protein was isolated and assessed using western blotting.

### 2.4. Animals

For this study, female Sprague Dawley rats were purchased from the Shanghai SLAC Laboratory Animal Co., Ltd. Initially, the rats were randomly divided into three groups (*n* = 6/group) as follows: (1) the control group, where the animals obtained no treatment and (2) the PCOS model-saline group, where 21-day-old female Sprague Dawley rats were subcutaneously injected with dehydroepiandrosterone (DHEA, 6 mg per 100 g body weight in 0.2 ml sesame oil) every day for a total of 21 days to induce hyperandrogenic PCOS [17, 18]. The rats were then intraperitoneally injected with 1 ml/kg saline for a subsequent 28 days; (3) the PCOS model–SYVN1 inhibitor (LS-102) group, where the rats were induced with DHEA to have the PCOS phenotype as mentioned in the previous group. Then, the rats were intraperitoneally injected with LS-102 (50 mg/kg) every day for a total of 28 days.

All the animal experiments were approved by the Shanghai East Hospital and were carried out in accordance with the regulations set by the Shanghai East Hospital. Ethical clearance was obtained from the ethics committee of the Shanghai East Hospital.

### 2.5. Plasmid and Small Interfering RNA Construction

To develop the SYVN1 (NM_032431.3) overexpression plasmid, the coding sequence of SYVN1 was initially amplified by PCR using primers 5′-ATGTTCCGCACGGCAGTGATG-3′ and 5′-TCAGTGGGCAACAGGAGACTCC-3′. Then, the amplified regions were cloned into a pcDNA3.1 plasmid. The siRNAs specifically targeting SYVN1 (GCTCTTTCACTGCCGCATT) and Drp1 (ACTCAAGACACCTTTCTAA) were designed and synthesized by GenePharma (Shanghai, China).

### 2.6. Quantitative Real-Time PCR

TRIzol reagent was used to isolate the total RNA from the granulosa cell samples (Invitrogen). Using HiScript reverse transcriptase, the total RNA was used in reverse transcription (RT) PCR to obtain cDNA (RNase H; Genecopoeia, Rockville, MD, USA). Finally, relative SYVN1 gene expression was assessed using the step one plus real-time PCR system (Applied Biosystem, Waltham, MA, USA). The primer sequences used are listed as follows: forward, 5′-CTCCTCCATCCACCAGTGC-3′ and reverse, 5′-GTGGTTGTAGCTGCTCCACT-5′. The relative gene expression was assessed by the 2-*ΔΔ*Ct method. *β*-Actin was used as a control.

### 2.7. Immunoprecipitation

Immunoprecipitation was performed using magnetic beads and a Pierce MS-Compatible magnetic IP Kit (Pierce, Rockford, IL, USA) according to the manufacturer's protocol. Briefly, cells were collected and washed with ice-cold PBS and lysed directly in lysis buffer supplemented with fresh protease inhibitors (Sigma-Aldrich). After incubation on ice for 30 min, lysates were spun at 12000 rpm to clear cell debris and immunoprecipitated with antibody-coupled magnetic beads overnight at 4°C. After magnetic separation, the immunoprecipitated fraction was eluted and subjected to western blot analysis.

### 2.8. Western Blot Analysis

To obtain total cellular protein, cells and rat ovaries were treated with RIPA buffer containing protease inhibitors. The total protein was further quantified using the BCA method (Pierce, Rockford, IL, USA). A 10% SDS–polyacrylamide gel was used to separate 20 *μ*g of total protein by electrophoresis. The proteins were transferred onto PVDF membranes (Millipore, Bedford, MA, USA). The membranes were further blocked with 5% nonfat milk and subsequently incubated with rabbit monoclonal SYVN1 antibody (Abcam, ab170901; 1 : 1,000), rabbit polyclonal cleaved-caspase-3 (Abcam, ab49822, 1 : 500), rabbit polyclonal Bax (Sigma-Aldrich, B3428, 1 : 500), rabbit polyclonal Bcl-2 (Abcam, ab196495, 1 : 500), mouse monoclonal Drp1 (Abcam, ab156951, 1 : 1,000), and rabbit polyclonal Mnf1 (Sigma-Aldrich, SAB2106161, 1 : 1,000), overnight at 4°C. Next, the membranes were incubated with respective peroxidase-conjugated secondary antibody for 1 h. Proteins bands were finally detected using an enhanced chemiluminescent detection system (Immunoblot, 23225; Millipore, Billerica, MA, USA). Band intensity of the target protein was obtained using ImageJ (NIH, USA) and normalized with that of the mouse monoclonal *β*-actin (Abcam, ab8224, 1 : 1,000).

### 2.9. Immunofluorescence Staining

GCs were cultured on sterilized glass coverslips and were fixed with 4% paraformaldehyde (PFA). The coverslips were blocked with 0.1% BSA in PBS. Then, the cells were incubated with primary rabbit monoclonal SYVN1 antibody (1 : 500; Abcam) in PBS at 4°C overnight. Next, the cells were incubated with goat anti-rabbit Alexa Fluor 680-conjugated secondary antibody (1 : 500, Abcam) for 1 h. Finally, slides were mounted in mounting media (Vector Laboratories), and images were captured with a fluorescence microscope (Olympus, Tokyo, Japan).

### 2.10. TUNEL Assay

Human granulosa cells and KGN cells were cultured on glass coverslips and fixed using 4% PFA. Apoptotic cell analysis was performed using the terminal deoxynucleotidyl transferase-mediated dUTP nick-end labeling (TUNEL) staining kit (Roche, Mannheim, Germany) according to the manufacturer's instructions. DAPI was used to counterstain the nuclei. Imaging of the TUNEL-positive cells, which have a pyknotic nucleus with dark red fluorescent staining, was performed using an inverted microscope.

### 2.11. Flow Cytometry Assay

KGN cells in each group were initially trypsinized and collected through centrifugation. These cells were washed three times with PBS and suspended in 100 *μ*L of binding buffer. The cells were incubated with 5 *μ*L of annexin V and 5 *μ*L of propidium iodide (PI). The mixture was incubated in dark for 15 min. The samples were then analyzed using flow cytometry. With annexin V at the *X* axis and PI on the *Y* axis, the left lower quadrant in the cytogram indicated normal cells, whereas the left upper quadrant indicated the cells that were injured mechanically. Further, the right lower quadrant represented early apoptotic cells, and the right upper quadrant represented advanced apoptotic or late necrotic cells.

### 2.12. Mitochondrial Morphology Assay

The cells were incubated with MitoTracker Red (Invitrogen, 20 nM) for 20 min, and then, mitochondrial images were taken using confocal microscopy.

### 2.13. Histological Assay and Follicle Count

Rat ovaries and vital organs from respective groups were initially fixed in 10% neutral-buffered formalin solution for 24 h at 4°C. The organs were dehydrated, embedded in paraffin, and sectioned serially at 5 *μ*m thickness. The sections were carefully mounted onto a frosted glass slide for further staining with hematoxylin and eosin (HE). Imagining of the sections was performed with a bright field microscope. Follicle number was evaluated under a microscope in every tenth HE-stained section to create a distance of approximately 50–60 *μ*m between counts. The follicles were categorized as primordial, primary, antral, Graafian, and atretic follicles according to the definitions described by Furat Rencber et al. [19].

### 2.14. Transmission Electron Microscopy (TEM)

Rat ovarian tissues were initially fixed for 2 h with 2.5% glutaraldehyde (Sigma-Aldrich). Tissues were washed three times in 0.1 M PBS, dehydrated in graded alcohol, and stained with OsO_4_ and uranyl acetate. The tissues were embedded into Durcupan Fluka resin and polymerized for 48 h at 56°C. Blocks were further sectioned into 55 nm ultrathin-section and collected on Formvar-coated single-slot grids. Sections were subsequently stained by incubating in lead citrate. The cell ultrastructure was observed using a transmission electron microscope (FEI, Hillsboro, OR, USA).

### 2.15. Statistical Analysis

Statistical analysis was performed using SPSS software version 17.0 (SPSS, Inc., Chicago, IL, USA). Data are presented as the mean ± standard deviation (SD). For normally distributed data, group differences were assessed using the Student *t*-test (comparison of two independent groups), one-way analysis of variance (ANOVA) with Tukey's multiple comparison test, or two-way ANOVA with Sidak's multiple comparison test (comparison of several independent groups). *P* value < 0.05 was considered to indicate a statistically significant difference.

## 3. Results

### 3.1. Downregulated SYVN1 Is Potentially Associated with Massive Apoptosis in Granulosa Cells of Polycystic Ovary Syndrome Patients

**Figure 1:**
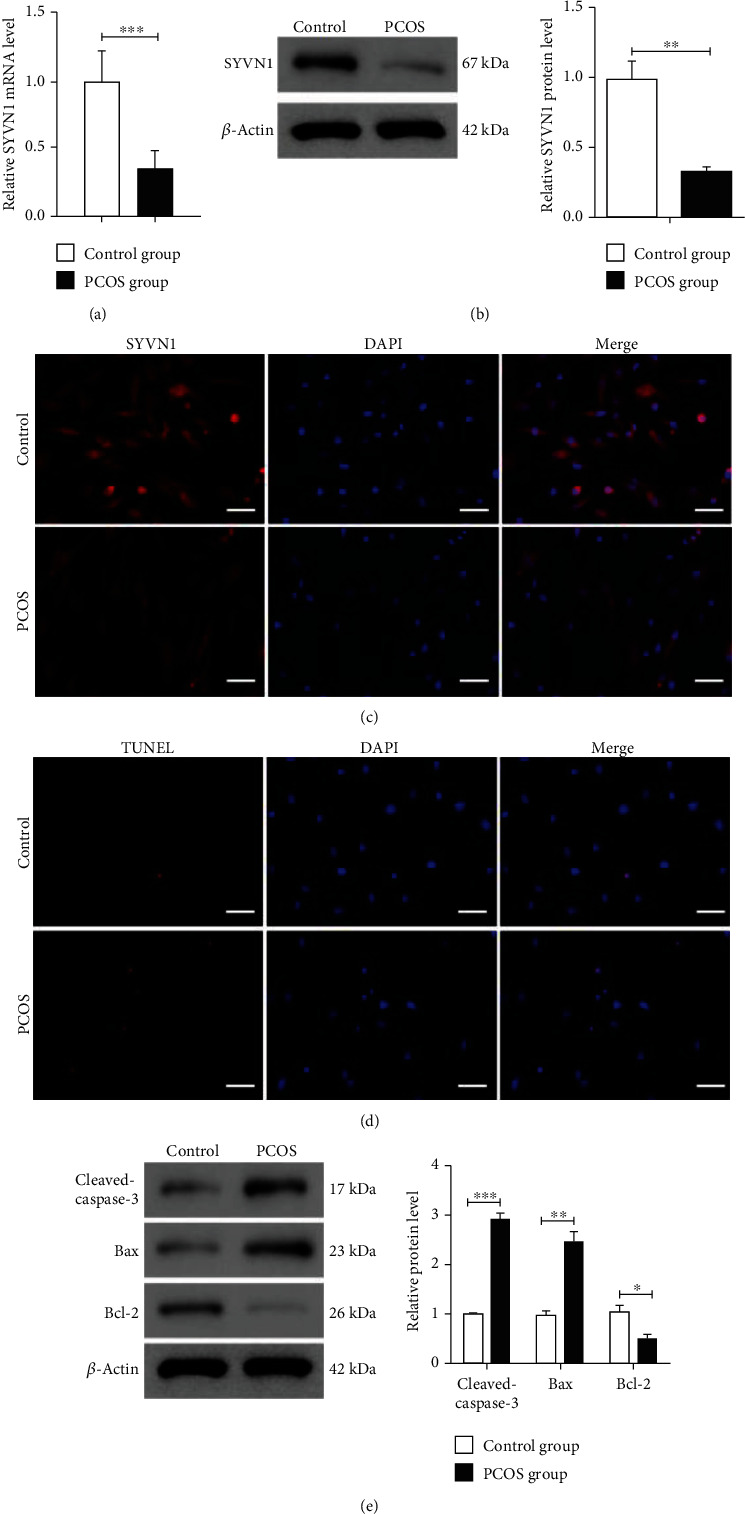
Downregulated SYVN1 is potentially associated with massive apoptosis in granulosa cells of PCOS patients. (a) The mRNA expression of SYVN1 was detected by RT-qPCR (*n* = 30). ^∗∗∗^*P* < 0.001, two-tailed unpaired Student's *t*-test. *P* < 0.0001, *t* = 13.43, and df = 58. (b) The protein levels of SYVN1 were detected by western blotting (*n* = 5). ^∗∗^*P* < 0.01, two-tailed unpaired Student's *t*-test. *P* = 0.0011, *t* = 4.997, and df = 8. (c) The expression of SYVN1 was detected by immunofluorescence. Red staining indicates SYVN1, and blue staining indicates the nucleus (DAPI). Scale bar = 50 *μ*m. (d) Granulosa cell apoptosis was detected by TUNEL. Scale bar = 50 *μ*m. (e) Protein expression of cleaved-caspase-3, Bax, and Bcl-2 of ovarian granulosa cells in each group was detected by western blotting. (control: patients undergoing fertility treatment due to male infertility or tubal infertility disorders; PCOS, patients with polycystic ovary syndrome). The data are expressed as mean ± standard deviation. (*n* = 4) ^∗^*P* < 0.05, ^∗∗^*P* < 0.01, and ^∗∗∗^*P* < 0.001. Two-tailed unpaired Student's *t*-test. Cleaved-caspase-3: *P* < 0.0001, *t* = 16.64, and df = 6; Bax: *P* = 0.0011, *t* = 5.836, and df = 6; Bcl-2: *P* = 0.0100, *t* = 3.705, and df = 6.

Initially, we assessed the mRNA expression levels of SYVN1 in primary GCs from patients with PCOS and identified that SYVN1 levels were significantly downregulated in PCOS patient cells (*n* = 30) when compared with control samples (Figure 1(a)). We also observed a significant decrease in SYVN1 protein levels in PCOS patient GCs (Figure 1(b)). We further visualized this decrease in PCOS GCs using immunofluorescence staining (Figure 1(c)). We observed an increase in the number of TUNEL-positive apoptotic cells among the PCOS GCs (Figure 1(d)). To further explore the role of apoptosis in PCOS, we checked the levels of key apoptotic markers such as cleaved caspase-3, Bax, and Bcl-2 using western blotting. We found a significant increase in the levels of cleaved caspase-3 and Bax, whereas a clear decrease in the Bcl-2 levels among PCOS GC samples (Figure 1(e)). This evidence indicates the potential involvement of SYVN1 in the apoptosis of GCs in PCOS patients.

### 3.2. Overexpression of SYVN1 Inhibited Apoptosis in KGN Cells

**Figure 2:**
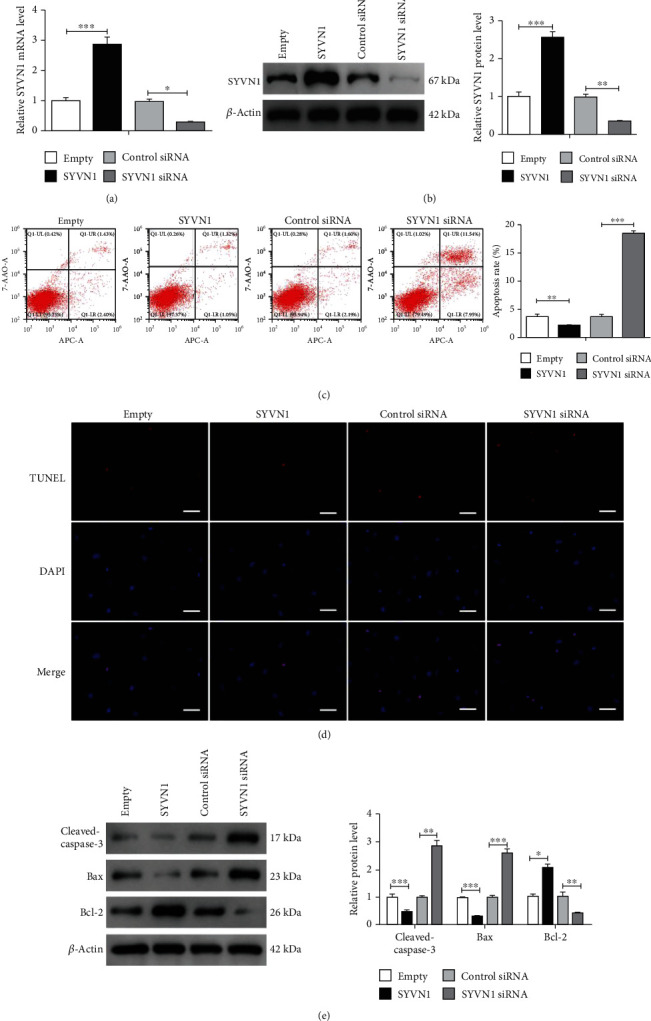
Overexpression of SYVN1 inhibited apoptosis in KGN cells. KGN cells were transfected with empty, SYVN1, control siRNA, or SYVN1 siRNA plasmids for 24 h. (a, b) RT-qPCR and western blot analysis for SYVN1 to test the transfection efficiency for SYVN1 overexpression or inhibition. ^∗^*P* < 0.05, ^∗∗^*P* < 0.01, and ^∗∗∗^*P* < 0.001. One-way ANOVA with Tukey's multiple comparison test. (a) *F* (3, 8) = 65.04, *P* < 0.0001. *P*_empty vs.SYVN1_ < 0.0001, *P*_control siRNA vs.SYVN1 siRNA_ = 0.0319; (b) *F* (3, 8) = 89.42, *P* < 0.0001. *P*_empty vs.SYVN1_ < 0.0001, *P*_control siRNA vs.SYVN1 siRNA_ = 0.0083. (c) Apoptosis of KGN cells in each group was detected by flow cytometry. Cartogram of apoptosis of KGN cells in each group. ^∗∗^*P* < 0.01 and ^∗∗∗^*P* < 0.001. One-way ANOVA with Tukey's multiple comparison test. *F* (3, 8) = 593.1, *P* < 0.0001. *P*_empty vs.SYVN1_ = 0.0017, *P*_control siRNA vs.SYVN1 siRNA_ < 0.0001. (d) Apoptosis of KGN cells in each group was measured by TUNEL staining. Scale bar = 50 *μ*m. (e) Protein expression of cleaved-caspase-3, Bax, and Bcl-2 in ovarian granulosa cells from each group was detected by western blotting. The data are expressed as mean ± standard deviation. ^∗^*P* < 0.05, ^∗∗^*P* < 0.01, and ^∗∗∗^*P* < 0.001. One-way ANOVA with Tukey's multiple comparison test. Cleaved-caspase-3: *F* (3, 8) = 65.81, *P* < 0.0001. *P*_empty vs.SYVN1_ < 0.0001, *P*_control siRNA vs.SYVN1 siRNA_ = 0.0078; Bax: *F* (3, 8) = 118.6, *P* < 0.0001. *P*_empty vs.SYVN1_ < 0.0001, *P*_control siRNA vs.SYVN1 siRNA_ = 0.0027; Bcl-2: *F* (3, 8) = 34.73, *P* < 0.0001. *P*_empty vs.SYVN1_ = 0.0279, *P*_control siRNA vs.SYVN1 siRNA_ = 0.0011.

To understand the role of SYVN1 in the apoptosis of GCs in PCOS, we used the human ovarian granulosa-like tumor (KGN) cell line. We overexpressed SYVN1 using expression plasmids, silenced SYVN1 using siRNA in the KGN line, and confirmed its expression levels using RT-qPCR and western blotting (Figures 2(a) and 2(b)). Apoptosis levels were assessed using flow cytometry. SYVN1 overexpression decreased the percentage of apoptotic cells (Empty: 3.83% and SYVN1: 2.37%), whereas silencing of SYVN1 increased the percent of apoptotic cells (siSYVN1: 12.94%) (Figure 2(c)). TUNEL assay indicated that SYVN1 overexpression decreased apoptotic TUNEL^+^ cells whereas silencing SYVN1 increased TUNEL^+^ cells (Figure 2(d)). We also confirmed the levels of apoptotic markers using western blotting. Overexpression of SYVN1 levels significantly decreased cleaved caspase and Bax levels along with an increase in the Bcl-2 levels. Alternatively, silencing of SYVN1 significantly increased cleaved caspase and Bax levels with a decrease in Bcl-2 levels (Figure 2(e)). These results indicate that SYVN1 regulates apoptosis in ovarian GCs.

### 3.3. Overexpression of SYVN1 Inhibited Mitochondrial Fission in KGN Cells

**Figure 3:**
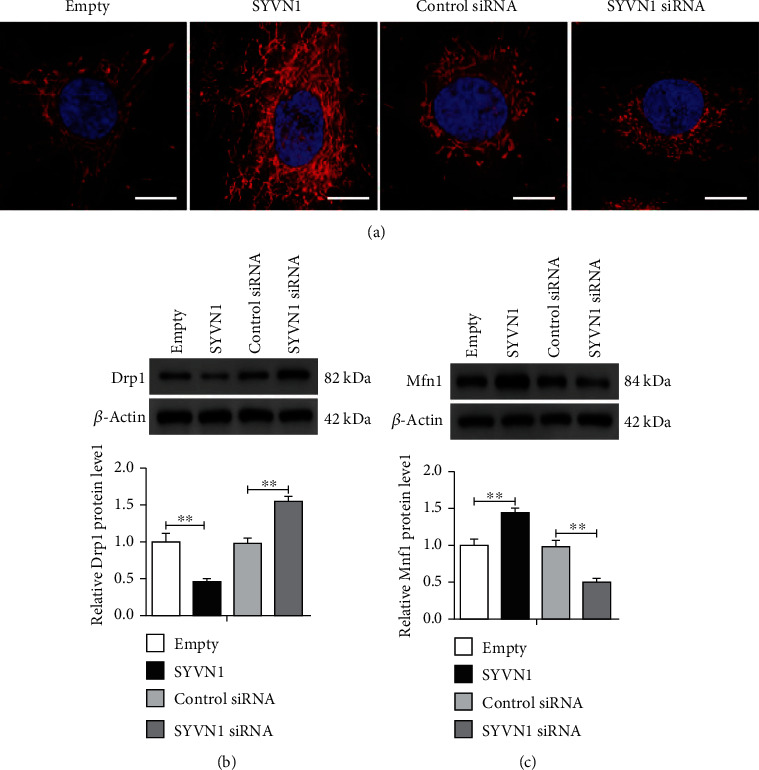
Overexpression of SYVN1 inhibited mitochondrial fission in KGN cells. KGN cells were transfected with empty, SYVN1, control siRNA, or SYVN1 siRNA plasmids for 24 h. (a) Imaging of mitochondrial morphology in each group by confocal microscopy using MitoTracker Red. Scale bar = 10 *μ*m. (b, c) Protein expression of Drp1 and Mfn1 in each group was detected by western blotting. The data are expressed as mean ± standard deviation. ^∗∗^*P* < 0.01. One-way ANOVA with Tukey's multiple comparison test. (b) *F* (3, 8) = 34.15, *P* < 0.0001. *P*_empty vs.SYVN1_ = 0.0042, *P*_control siRNA vs.SYVN1 siRNA_ = 0.0032; (c) *F* (3, 8) = 31.44, *P* < 0.0001. *P*_empty vs.SYVN1_ = 0.0090, *P*_control siRNA vs.SYVN1 siRNA_ = 0.0045.

As mitochondrial fission is the initial event during apoptosis, we wanted to explore the role of SYVN1 in mitochondria. Using MitoTracker, we assessed KGN cells either overexpressing or silenced for SYVN1. Imaging results indicated that the silencing of SYVN1 significantly decreased the mitochondrial levels as indicated through decreased surface area exhibiting a positive signal for MitoTracker. Alternatively, SYVN1 overexpression clearly increased the mitochondrial signal (Figure 3(a)). Overexpression of SYVN1 showed highly interconnected and elongated mitochondrial structures, whereas silencing of SYVN1 resulted in stratified, disconnected, and circular mitochondria. These results indicate that SYVN1 could play a role in apoptosis by inhibiting mitochondrial fission. Next, we assessed the levels of key the mitochondrial fission-associated protein Drp1 in KGN cells. Initially, it was clear that overexpression of SYVN1 significantly decreased Drp1 levels, whereas silencing of SYVN1 increased Drp1 (Figure 3(b)). We also checked the levels of mitofusin 1 (Mfn1) and observed that overexpression of SYVN1 significantly increased Mfn1 levels, whereas silencing SYVN1 decreased Mfn1 levels (Figure 3(c)). This further confirmed that SYVN1 inhibited mitochondrial fission.

### 3.4. SYVN1 Regulates Ubiquitination and Proteasomal Degradation of Drp1 in KGN Cells

**Figure 4:**
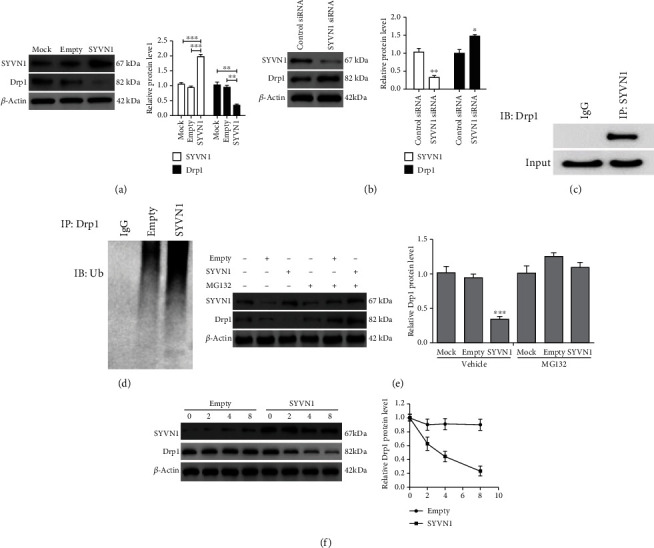
SYVN1 controls ubiquitination and proteasomal degradation of Drp1 in KGN cells. (a) KGN cells were either transfected or not transfected with empty or SYVN1 overexpression plasmids, and western blotting was performed to examine protein expression of SYVN1 and Drp1 (*n* = 3). ^∗∗^*P* < 0.01 and ^∗∗∗^*P* < 0.001. One-way ANOVA with Tukey's multiple comparison test. SYVN1: *F* (2, 6) = 75.35, *P* < 0.0001. *P*_Mock vs.SYVN1_ = 0.0001, *P*_empty vs.SYVN1_ < 0.0001; Drp1: *F* (2, 6) = 24.44, *P* = 0.0013. *P*_Mock vs.SYVN1_ = 0.0017, *P*_empty vs.SYVN1_ = 0.0031. (b) Western blot analysis of the expression of SYVN1 and Drp1 in KGN cells transfected with control siRNA or SYVN1 siRNA (*n* = 3). ^∗^*P* < 0.05 and ^∗∗^*P* < 0.01. Two-tailed unpaired Student's *t*-test. SYVN1: *P* = 0.0049, *t* = 5.625, df = 4; Drp1: *P* = 0.0183, *t* = 3.843, df = 4. (c) SYVN1 binding to Drp1 was detected by immunoprecipitation. (d) Western blotting was used to detect the effect of SYVN1 on Drp1 ubiquitin modification. (e) Western blot analysis of the expression of Drp1 in KGN cells transfected with either empty or SYVN1, in the presence or absence of the proteasome inhibitor MG132 (*n* = 3). All blots used *β*-actin as loading controls. ^∗∗∗^*P* < 0.001. Two-way ANOVA with Sidak's multiple comparisons test. *F* (5, 10) = 31.29, *P* < 0.0001. (f) A cycloheximide (CHX) chase assay was performed to assess the half-life of Drp1. KGN cells were transfected with empty or SYVN1 for 24 h. Cells were then treated with CHX (100 *μ*g/ml) for the indicated hours, and western blotting was performed. Relative Drp1 protein levels in KGN cells were quantified and plotted with the length (days) of CHX treatment on the *X* axis. The data are expressed as mean ± standard deviation.

To understand the role of SYVN1 in mitochondrial fission, we overexpressed SYVN1 and assessed Drp1 levels using western blotting. Our data clearly indicate that the overexpression of SYVN1 caused a significant decrease in Drp1 protein levels (Figure 4(a)). Additionally, silencing of SYVN1 significantly increased Drp1 protein levels (Figure 4(b)). We wanted to assess whether SYVN1 regulates Drp1 protein through ubiquitination. Therefore, we examined whether SYVN1 was able to bind Drp1 in Co-IP experiments. In KGN cells, SYVN1 was able to bind to Drp1 (Figure 4(c)), and the ubiquitination of Drp1 was detected using an ubiquitination antibody in KGN cells overexpressing SYVN1 (Figure 4(d)). To confirm that SYVN1 was able to regulate the protein level of Drp1 through the proteasome pathway, we treated SYVN1 overexpressed cells with the proteasome inhibitor MG132, and the results showed that MG132 could indeed inhibit the downregulation of Drp1 level by SYVN1 (Figure 4(e)). Meanwhile, treatment of KGN cells with CHX revealed that the overexpression of SYVN1 was able to significantly reduce Drp1 stability (Figure 4(f)). These results showed that in KGN cells, the binding of SYVN1 to Drp1 promoted its ubiquitination and degradation.

### 3.5. SYVN1 Inhibited Apoptosis and Mitochondrial Fission by Promoting Drp1 Degradation in KGN Cells

**Figure 5:**
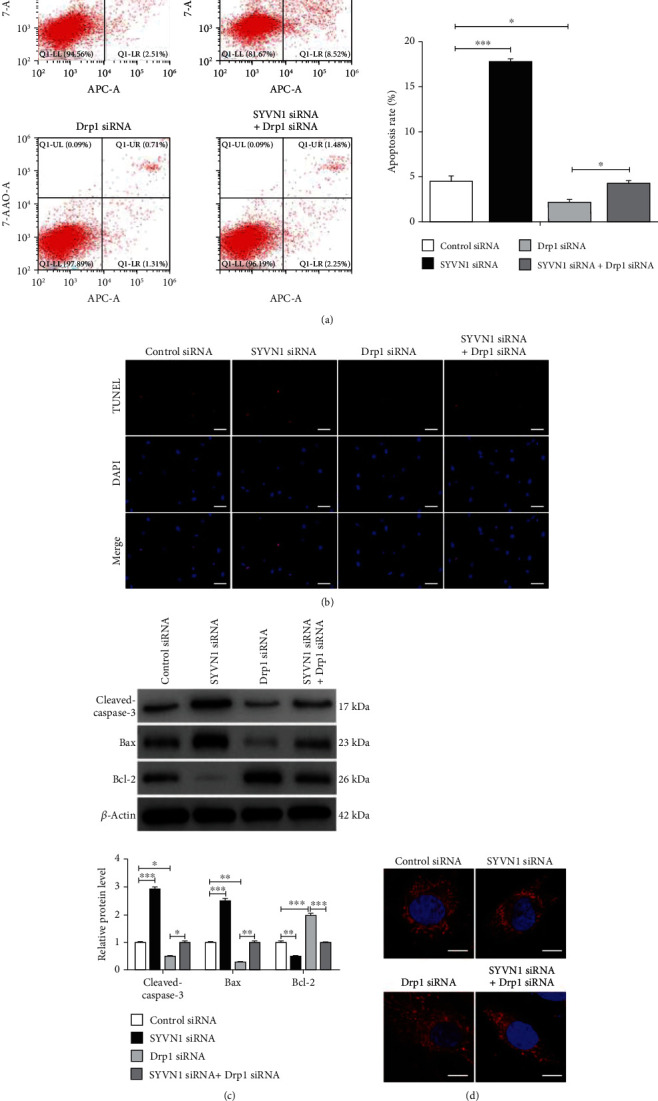
SYVN1 inhibited apoptosis and mitochondrial fission by promoting Drp1 degradation in KGN cells. KGN cells were transfected with control siRNA, SYVN1 siRNA, Drp1 siRNA, or SYVN1 siRNA+Drp1 siRNA for 24 h. (a) Apoptosis of KGN cells in each group was detected by flow cytometry. Cartogram of cell apoptosis in each group. ^∗^*P* < 0.05 and ^∗∗∗^*P* < 0.001. One-way ANOVA with Tukey's multiple comparison test. *F* (3, 8) = 230.3, *P* < 0.0001. *P*_control siRNA vs.SYVN1 siRNA_ < 0.0001, *P*_control siRNA vs.Drp1 siRNA_ = 0.0258, *P*_Drp1 siRNA vs.SYVN1 siRNA_ = 0.0286. (b) Apoptosis in each group of KGN cells was measured by TUNEL staining. Scale bars are 50 *μ*m. The percentages of TUNEL-positive cells are shown. (c) Protein expression of cleaved-caspase-3, Bax, and Bcl-2 of ovarian granulosa cells in each group. ^∗^*P* < 0.05, ^∗∗^*P* < 0.01, and ^∗∗∗^*P* < 0.001. One-way ANOVA with Tukey's multiple comparison test. Cleaved caspase-3: *F* (3, 8) = 203.3, *P* < 0.0001. *P*_control siRNA vs.SYVN1 siRNA_ < 0.0001, *P*_control siRNA vs.Drp1 siRNA_ = 0.0116, *P*_Drp1 siRNA vs.SYVN1 siRNA_ = 0.0112; Bax: *F* (3, 8) = 164.5, *P* < 0.0001. *P*_control siRNA vs.SYVN1 siRNA_ < 0.0001, *P*_control siRNA vs.Drp1 siRNA_ = 0.0012, *P*_Drp1 siRNA vs.SYVN1 siRNA_ = 0.0010; Bcl-2: *F* (3, 8) = 80.24, *P* < 0.0001. *P*_control siRNA vs.SYVN1 siRNA_ = 0.0072, *P*_control siRNA vs.Drp1 siRNA_ < 0.0001, *P*_Drp1 siRNA vs.SYVN1 siRNA_ < 0.0001. (d) Imaging mitochondrial morphology in each group by confocal microscopy and MitoTracker Red. Scale bars are 10 *μ*m. Changes in mitochondrial morphology were quantified by the ImageJ software. The data are expressed as mean ± standard deviation.

The role that SYVN1 played in the regulation of apoptosis was characterized by silencing SYVN1 and Drp1. Flow cytometry showed that the silencing of SYVN1 increased apoptosis in KGN cells, whereas the silencing of Drp1 gave the opposite result and decreased apoptosis in KGN cells. In addition, silencing Drp1 reversed apoptosis induced by SYVN1 knockout, increasing the level of apoptosis (Figure 5(a)). We confirmed these results using a TUNEL assay and observed that SYVN1 is necessary for the decrease in Drp1-mediated apoptosis (Figure 5(b)). Western blotting also indicated that the silencing of SYVN1 caused a significant increase in apoptotic markers (Bax and cleaved caspase-3). However, silencing of Drp1 significantly decreased Bax and cleaved caspase levels. In parallel, we observed a decrease in Bcl-2, when SYVN1 was silenced, and an increase in Bcl-2, when Drp1 was silenced (Figure 5(c)). We also assessed mitochondrial fission levels using MitoTracker, and it was clear that silencing of SYVN1 increased mitochondrial fission. In contrast, silencing of Drp1 significantly decreased mitochondrial fission as indicated by an increase in the mitochondrial surface area along with highly interconnected and elongated mitochondrial structures. However, the silencing of Drp1 along with SYVN1 reversed this effect and resulted in rarefied and disconnected mitochondrial structures (Figure 5(d)). These results confirmed that SYVN1 decreased apoptosis and mitochondrial fission by inducing the degradation of Drp1.

### 3.6. SYVN1 Inhibited Apoptosis and Mitochondrial Fission in Granulosa Cells in Rat with Polycystic Ovary Syndrome

**Figure 6:**
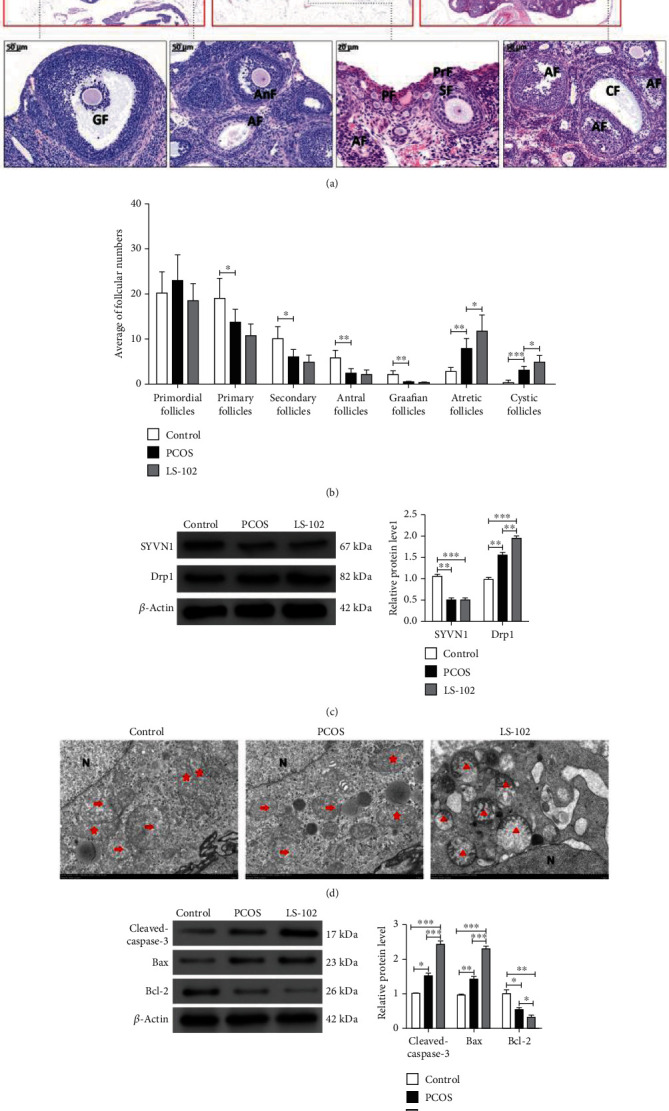
SYVN1 inhibited apoptosis and mitochondrial fission in granulosa cells in rats with polycystic ovary syndrome. Female Sprague Dawley rats were injected subcutaneously with DHEA for 21 days and injected intraperitoneally with saline or SYVN1 inhibitor (LS-102) for 28 days. (a) Ovarian histological staining was performed in each group using H&E. PF: primordial follicles; PrF: primary follicles; SF: secondary follicles; AnF: antral follicles; GF: Graafian follicles; AF: atretic follicles; CF: cystic follicle cysts. (b) Average number of primordial, primary, secondary, antral, Graafian follicles, atretic follicles, and cystic follicles in control, PCOS, and LS-102-treated rats. ^∗^*P* < 0.05, ^∗∗^*P* < 0.01, and ^∗∗∗^*P* < 0.001. One-way ANOVA with Tukey's multiple comparison test. Primordial follicles: *F* (2, 15) = 1.225, *P* = 0.3216. Primary follicles: *F* (2, 15) = 9.075, *P* = 0.0026. *P*_control vs.PCOS_ = 0.0418; secondary follicles: *F* (2, 15) = 10.45, *P* = 0.0014. *P*_control vs.PCOS_ = 0.0105; antral follicles: *F* (2, 15) = 14.09, *P* = 0.0004. *P*_control vs.PCOS_ = 0.0011; Graafian follicles: *F* (2, 15) = 15.42, *P* = 0.0002. *P*_control vs.PCOS_ = 0.0013; atretic follicles: *F* (2, 15) = 20.01, *P* < 0.0001. *P*_control vs.PCOS_ = 0.0076; cystic follicles: *F* (2, 15) = 31.56, *P* < 0.0001. *P*_control vs.PCOS_ = 0.0008, *P*_PCOS vs.LS−102_ = 0.0155. (c) The protein levels of SYVN1 and Drp1 were detected in each group by western blotting. ^∗∗^*P* < 0.01 and ^∗∗∗^*P* < 0.001. One-way ANOVA with Tukey's multiple comparison test. SYVN1: *F* (2, 6) = 21.35, *P* = 0.0019. *P*_control vs.PCOS_ = 0.0032, *P*_control vs.LS−102_ = 0.0031; Drp1: *F* (2, 6) = 57.77, *P* = 0.0001. *P*_control vs.PCOS_ = 0.0028, *P*_control vs.LS−102_ < 0.0001, *P*_PCOS vs.LS−102_ = 0.0061. (d) Representative transmission electron microscopy images of the mitochondrial network in ovarian tissues from each group. Asterisks, arrows, and triangles indicate elongated, intermediate (mid), and fragmented mitochondria, respectively. N: nucleus. Scale bar: 2 mm. (e) Protein expression of cleaved-caspase-3, Bax, and Bcl-2 of ovarian granulosa cells in each group was detected by western blotting. The data are expressed as mean ± standard deviation. ^∗^*P* < 0.05, ^∗∗^*P* < 0.01, and ^∗∗∗^*P* < 0.001. One-way ANOVA with Tukey's multiple comparison test. Cleaved caspase-3: *F* (2, 6) = 77.00, *P* < 0.0001. *P*_control vs.PCOS_ = 0.0110, *P*_control vs.LS−102_ < 0.0001, *P*_PCOS vs.LS−102_ = 0.0005; Bax: *F* (2, 6) = 147.4, *P* < 0.0001. *P*_control vs.PCOS_ = 0.0024, *P*_control vs.LS−102_ < 0.0001, *P*_PCOS vs.LS−102_ < 0.0001; Bcl-2: *F* (2, 6) = 18.57, *P* = 0.0027. *P*_control vs.PCOS_ = 0.0162, *P*_control vs.LS−102_ < 0.0024, *P*_PCOS vs.LS−102_ = 0.0494 (control group: untreated rats; PCOS group: saline was injected through the abdominal cavity of the PCOS model rats. LS-102 group: LS-102 was injected through the abdominal cavity of the PCOS model rats).

Finally, we assessed the role of SYVN1 in inhibiting apoptosis and mitochondrial fission in a rat model of PCOS. We separated the female SD rats (*N* = 18) into two groups, a control (*N* = 6) and PCOS model groups (*N* = 12). To achieve a PCOS model, we injected rats intraperitoneally with DHEA for 21 days. These rats were again divided into two groups, and one group was assigned as the control and was injected with saline for 28 days (PCOS, *N* = 6). The other group was injected with LS-102, an SYVN1 inhibitor, for 28 days (LS-102, *N* = 6). After 28 days, the rats were euthanized, organs were extracted, and total protein was isolated. Using HE staining, we confirmed the proper establishment of PCOS as indicated by the degeneration of oocyte and GC layer, along with the presence of large follicles in the PCOS rat ovaries. We observed that the LS-102 aggravated the PCOS phenotype, with an increased number of large fluid-filled follicles and increased degeneration of oocytes posttreatment (Figure 6(a)). We further investigated the consequences of PCOS on follicle morphology by assessing the different follicles in each ovary (Figure 6(b)). The number of primary, secondary, antral, and Graafian follicles was significantly reduced in the PCOS group, whereas the number of atretic and cystic follicles was significantly increased. Compared to the PCOS group, the number of primordial, primary, secondary, antral, and Graafian follicles in the LS-102-treated group essentially remained unchanged (*P* < 0.05). However, the number of atretic and cystic follicles was significantly increased. This indicates that SYVN1 may play potential roles in follicular development. The samples were assessed for SYVN1 and Drp1 protein levels using western blotting. We observed that the DHEA-induced PCOS groups (saline and LS-102 treated) had significantly lower levels of SYVN1 when compared to the control group (Figure 6(c)). A higher number of fragmented mitochondria were observed in the PCOS and LS-102 group when compared to the control group (Figure 6(d)). Western blotting indicated that the PCOS group displayed significantly high levels of cleaved caspase and Bax, but a significantly low level of Bcl-2 when compared to the control group. LS-102 treatment elevated the levels of apoptosis as indicated by increased cleaved caspase-3 and Bax levels, along with decreased Bcl-2 levels, compared to both control and PCOS groups (Figure 6(e)). These results indicated that SYVN1 plays an important role in inhibiting apoptosis and mitochondrial fission in the GC cells of rats with PCOS.

## 4. Discussion

Women with PCOS are considered at greater risk of many physiological and psychological long-term complications [2]. Genetics, oxidative stress, insulin resistance, lipid imbalance, and hyperinsulinemia have been identified as some of the contributing factors to PCOS [1, 20, 21]. However, the etiology of this disease is still unclear. The most common strategy used to alleviate PCOS is the use of drugs, such as metformin, that target metabolic and hormonal complications [22]. Alternative strategies use selenium to act as an antioxidant and to reduce the devastating consequences of ROS in PCOS [23]. A study by Olaniyan et al. suggested that ROS can contribute to the apoptosis of GCs, and the use of 3-nitropropionic acid could decrease ROS production and associated apoptosis [24]. In this study, we further examine the molecular pathway through which this apoptosis is caused in the GCs. Initially, using protein-protein interaction network analysis, we identified that SYVN1, the E3 ubiquitin ligase, could be associated with PCOS. We further confirmed that SYVN1 is significantly downregulated and is associated with increased apoptosis in PCOS granulosa and KGN cells. The increase in apoptosis was further characterized by cleaved caspase-3 and the proapoptotic marker Bax. Indeed, we observed an upregulation of these markers in PCOS cells and a clear decrease in antiapoptotic markers such as Bcl-2. Several reports on BCL-2 expression in PCOS have found decreases in the level of GCs in PCOS patients and in the ovarian tissues of PCOS animal models [25, 26], which correspond with the results found in our study. Overall, our results imply that ovarian GC cell apoptosis is essential for the progression of PCOS.

In this study, we also observed that apoptosis in PCOS and the downregulation of SYVN1 are highly correlated with increased mitochondrial fission. Mitochondrial dynamics are complex, and the regulation of the fusion and fission process is vital [4]. Mitochondria fuse and divide as they migrate along the cytoskeleton, fusion is followed by fission, and the absence or deregulation of either could be deleterious to the cell [4]. In this study, we were specifically interested in the role of mitochondrial fission in PCOS. Drp1 is considered a vital regulator of mitochondrial fission, and cells lacking Drp1 are known to have highly interwoven mitochondrial nest-like structures [27, 28]. Drp1, alternatively known as Dnm1 in yeast, functions through its interaction with its two partners, mitochondrial fission 1 (Fis1) and mitochondrial division protein 1 (Mdv1) [29, 30]. The process of mitochondrial fission is probably initiated by Fis1 which recruits Mdv1 to the mitochondrial membrane [4, 31]. Mdv1 nucleates the Dnm1/Drp1-GTP oligomer, which forms spirals around the mitochondrial membrane and serves the mitochondria into fragments [4]. Previous, studies have indicated that the upregulation of Fis1 leads to increased fragmentation of mitochondria, whereas downregulation of Fis1 leads to fused mitochondria [32].

In PCOS GCs, we observed that SYVN1 downregulation is associated with the upregulation of Drp1. Indeed, we could clearly observe an associated increase in mitochondrial fission and apoptosis in the PCOS models. Understanding the role of Drp1 in apoptosis would be essential to understanding its role in PCOS. Under apoptotic stimulus, Drp1 migrates to the mitochondrial membrane and recruits Mfn1 and Bax to initiate mitochondrial fission. Studies similar to ours [33, 34] have indicated that the lack of Drp1 could decrease mitochondrial fission, cleaved caspase-3 production, and apoptosis. Inhibition of Drp1 appears to inhibit the apoptotic process just before Bax translocation and cytochrome C release [35]. Hence, the regulation of Drp1 is a vital step in the regulation of apoptosis in PCOS. SYVN1 is an E3 ubiquitin ligase protein identified to play a vital role in ER-associated degradation [14]. Defects in ER-associated degradation have been associated with diseases where the disposal of accumulating toxic protein is necessary, such as Alzheimer's and Parkinson's [36].

Interestingly, SYVN1 has been associated with the ubiquitination of these toxic proteins and degrading them through the proteasomal pathway, thus maintaining the balance in the ER and decreasing ER-associated stress [37, 38]. SYVN1 is also known to mediate ubiquitination and degradation of proteins to regulate apoptosis in other diseases such as ER proteotoxic stress and liver injury [39] and lens epithelial cells [40]. Both of these diseases involve the misfolding and aggregation of proteins through SYVN1-mediated degradation via the ubiquitin-proteasome pathway. In this study, we identified that Drp1 coprecipitates with SYVN1 and that the upregulation of SYVN1 further degrades Drp1. Moreover, Drp1 degrades quickly in the presence of SYVN1. We also observed that the use of proteasomal inhibitor upregulated Drp1 levels even in the presence of SYVN1. Overall, our results indicate that SYVN1 regulates the levels of Drp1 using the proteasome/ubiquitin pathway.

In a DHEA-mediated PCOS rat model, SYVN1 is highly downregulated in PCOS and an SYVN1 inhibitor exacerbated the PCOS by significantly increasing Drp1 and apoptosis. In addition to increased apoptosis, we also found an elevated level of atretic follicles in the PCOS rat model and the number increased even further when Drp1 was upregulated by the use of protease inhibitor, implying that SYVN1 can influence folliculogenesis. In support of our results, Salehi et al. [12] observed that Drp1 is associated with excessive mitochondrial fission and apoptosis in GCs and found that follicular growth arrest is important in the pathogenesis of PCOS. They found that GC apoptosis and follicular growth arrest can disrupt folliculogenesis and anovulation, and that upregulated Drp1 can increase levels of mitochondrial fission. Overall, we obtained similar results, SYVN1 binds and degrades Drp1, and the loss of Drp1 thus modulates mitochondrial fission and apoptosis in ovarian GCs.

## 5. Conclusions

This study indicates that SYVN1 binds and degrades Drp1 and thus modulates mitochondrial fission and apoptosis in ovarian GCs. Moreover, the current study provides a novel target for the development of therapeutic strategies for PCOS.

## Data Availability

The datasets used and/or analyzed during the current study are available from the corresponding author on reasonable request.
